# Determinants of Phage Host Range in *Staphylococcus* Species

**DOI:** 10.1128/AEM.00209-19

**Published:** 2019-05-16

**Authors:** Abraham G. Moller, Jodi A. Lindsay, Timothy D. Read

**Affiliations:** a Program in Microbiology and Molecular Genetics (MMG), Graduate Division of Biological and Biomedical Sciences (GDBBS), Emory University School of Medicine, Atlanta, Georgia, USA; b Institute of Infection and Immunity, St. George’s, University of London, London, United Kingdom; c Division of Infectious Diseases, Department of Medicine, Emory University School of Medicine, Atlanta, Georgia, USA; University of Queensland

**Keywords:** CRISPR, host range, phage resistance, phage therapy, staphylococci

## Abstract

Bacteria in the genus *Staphylococcus* are important targets for phage therapy due to their prevalence as pathogens and increasing antibiotic resistance. Here we review *Staphylococcus* outer surface features and specific phage resistance mechanisms that define the host range, the set of strains that an individual phage can potentially infect. Phage infection goes through five distinct phases: attachment, uptake, biosynthesis, assembly, and lysis. Adsorption inhibition, encompassing outer surface teichoic acid receptor alteration, elimination, or occlusion, limits successful phage attachment and entry. Restriction-modification systems (in particular, type I and IV systems), which target phage DNA inside the cell, serve as the major barriers to biosynthesis as well as transduction and horizontal gene transfer between clonal complexes and species. Resistance to late stages of infection occurs through mechanisms such as assembly interference, in which staphylococcal pathogenicity islands siphon away superinfecting phage proteins to package their own DNA. While genes responsible for teichoic acid biosynthesis, capsule, and restriction-modification are found in most *Staphylococcus* strains, a variety of other host range determinants (e.g., clustered regularly interspaced short palindromic repeats, abortive infection, and superinfection immunity) are sporadic. The fitness costs of phage resistance through teichoic acid structure alteration could make staphylococcal phage therapies promising, but host range prediction is complex because of the large number of genes involved, and the roles of many of these are unknown. In addition, little is known about the genetic determinants that contribute to host range expansion in the phages themselves. Future research must identify host range determinants, characterize resistance development during infection and treatment, and examine population-wide genetic background effects on resistance selection.

## Abstract (precis)

Bacteria in the genus *Staphylococcus* are important targets for phage therapy due to their prevalence as pathogens and increasing antibiotic resistance. Here we review *Staphylococcus* outer surface features and specific phage resistance mechanisms that define the host range, the set of strains that an individual phage can potentially infect.

## INTRODUCTION

The *Staphylococcus* genus includes commensals and pathogens of humans and animals. Staphylococcus aureus and S. epidermidis, in particular, cause diverse infections in humans and have become increasingly antibiotic resistant over the past 70 years. Diseases range from food poisoning to skin and soft tissue infections, pneumonia, osteomyelitis, endocarditis, and septic shock. S. aureus is carried by between 20% (persistently) and 60% (intermittently) of the human population (1), primarily on the skin and upper respiratory tract. Methicillin-resistant S. aureus (MRSA) emerged in the mid-1960s (2), and methicillin resistance has reduced the options for treatment with beta-lactam antibiotics. The combination of high carriage rates, diverse pathologies, prevalent antimicrobial resistance, and a lack of a licensed vaccine (3) makes staphylococcal species important targets for new therapies.

Bacteriophages (phages) are natural killers of *Staphylococcus* bacteria, lysing bacterial cells through expression of holins, which permeabilize the membrane and release endolysins (4, 5) that degrade the peptidoglycan of the cell wall (6). Phage therapy is a promising alternative to antibiotics for treating infections because of the large number of diverse phages with low toxicity to humans and nontarget species (7, 8).

Phage therapy has a long history, reaching back before the antibiotic era to shortly after the discovery of phages themselves by Frederick Twort and Felix d’Herelle in the 1910s (9–11). While overshadowed by the subsequent discovery of antibiotics and generally abandoned in the West for many years, phage therapy persisted as a bacterial treatment in eastern Europe and the nations that composed the former Soviet Union (9, 10). There, phage cocktails were developed for the treatment of sepsis, osteomyelitis, and burn wounds, among other staphylococcal diseases, with complete recovery reported in some cases (12). Polish and Soviet studies showed that phage lysates effectively treated staphylococcal skin and lung infections (13). More recently, the emergence of multidrug resistance in bacterial pathogens renewed interest in phage therapy and phage biology (8, 14). Safety studies on the staphylococcal phage lysate (SPL) as well as phage cocktails containing S. aureus-specific phages indicated that they had no adverse effects when administered intranasally, intravenously, orally, topically, or subcutaneously (14). Phages have also been recently approved by the FDA as a treatment to clear another Gram-positive species (Listeria monocytogenes) present in food (15) and approved as personalized treatment for burn wound infections (16).

All known staphylococcal phages are members of the order *Caudovirales* with linear double-stranded DNA virion genomes. Staphylococcal phages are divided into three families with distinctive morphologies: the long, noncontractile-tailed *Siphoviridae*, the contractile-tailed *Myoviridae*, and the short, noncontractile-tailed *Podoviridae* (17, 18). *Siphoviridae* genomes are 39 to 43 kb in size, while those of the *Myoviridae* are 120 to 140 kb and those of the *Podoviridae* are 16 to 18 kb (17). Currently reported *Siphoviridae* are typically temperate phages that encode lysogeny functions within a genomic module, while reported *Myoviridae* and *Podoviridae* are virulent. The virulent phages are the strongest potential candidates for phage therapy, given that they are not known to lysogenize and, thus, obligately kill their targets. Lytic staphylococcal phages have surprisingly broad host ranges (19–22), antibiofilm activity (19, 23), and various degrees of effectiveness against infection (24–26). The *Siphoviridae* are agents of horizontal gene transfer (HGT) through transduction (27) into recipient strains (17) and activation of staphylococcal pathogenicity islands (SaPIs) (28). The *Siphoviridae* have been subdivided into integrase types based on the sequence of the integrase gene, necessary for lysogenic insertion into the chromosome (17, 29). Phages of certain integrase types introduce specific virulence factors (17). Integrase type 3 (Sa3int) phages encode the immune evasion cluster (IEC), which includes the staphylokinase (*sak*), staphylococcal complement inhibitor (*scn*), chemotaxis inhibitory protein (*chp*), and enterotoxin S (*sea*). In addition, Sa2int phages often encode Panton-Valentine leukocidin (*lukFS-PV*), while Sa1int phages often encode exfoliative toxin A (*eta*). Temperate staphylococcal phages can also disrupt chromosomal virulence factors (17). Sa3int and Sa6int phages, for example, integrate into sites in the beta-hemolysin (*hlb*) or lipase (*geh*) gene, respectively (30, 31).

No single phage can kill every *Staphylococcus* strain. Instead, each phage has a particular host range, defined as the set of strains permissive for its infection. Host range can be limited by active host resistance mechanisms, such as clustered regularly interspaced short palindromic repeat (CRISPR) or restriction-modification (R-M) systems that actively suppress phage infection, or by passive mechanisms, such as the loss of receptors for phage adsorption. It is unclear whether these host range-limiting factors have arisen through specific adaptation against phage infection or are by-products of selection against other stresses. There are, however, specific phage mechanisms counteracting host resistance that serve to broaden the phage host range. Phage host range has great importance to phage therapy because it defines the potential scope of treatable strains, thus informing the selection of phages for rational, personalized cocktail development.

**FIG 1:**
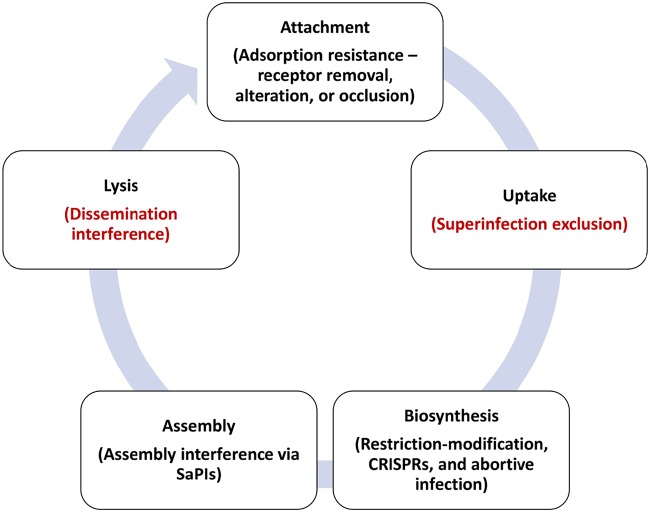
Stages of phage infection and corresponding examples of resistance mechanisms at each stage. Examples not yet identified in the staphylococci are listed in red.

Mechanisms of resistance to phages have previously been reviewed across bacteria generally (32, 33) and in lactic acid bacteria (34), but our article focuses on the particular features of *Staphylococcus* (Fig. 1). By far, the majority of the literature has focused on two species: S. epidermidis and, especially, S. aureus. However, we include studies on other species (e.g., S. simulans), where appropriate. We then reflect on the possible consequences of resistance on phage host range and potential phage therapy for staphylococcal infections, given that phage resistance elements determine host range and, thus, provide one criterion for phage efficacy in therapy. We also consider the evolutionary trade-offs of phage resistance in a therapeutic context due to the potential effects of phage resistance on either virulence or antibiotic resistance.

Host resistance can occur at different points in the phage life cycle (Fig. 1) (32, 33). There are no reports in *Staphylococcus* of mechanisms that limit the host range at the uptake and host lysis phases. We therefore concentrate on the attachment, biosynthesis, and assembly phases.

## ATTACHMENT

### Wall teichoic acid is the primary staphylococcal phage receptor.

**FIG 2:**
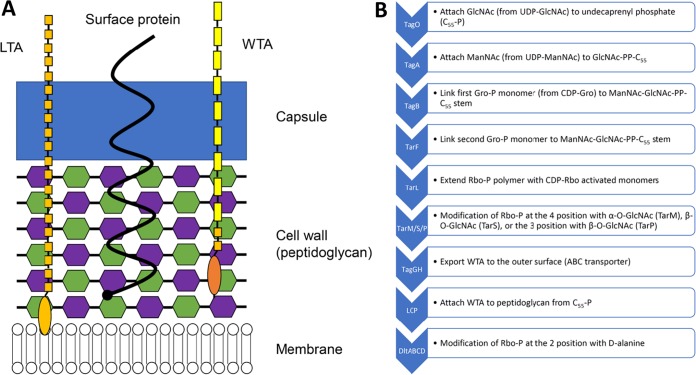
(A) Structure of the staphylococcal cell envelope. Lipoteichoic acid is shown in orange (glycerol phosphate), a surface protein is in black, wall teichoic acid is in orange (glycerol phosphate) and yellow (ribitol phosphate), capsule is in blue, and cell wall carbohydrates are in green (*N*-acetylglucosamine [GlcNAc]) and purple (*N*-acetylmuramic acid [MurNAc]). Staphylococcal phages bind WTA and/or its ribitol phosphate modifications (i.e., GlcNAc). (B) Outline of the wall teichoic acid (WTA) biosynthesis pathway, with the proteins corresponding to each step listed in the blue arrows. Abbreviations are defined as follows: C_55_-P, undecaprenyl phosphate; GlcNAc, *N*-acetylglucosamine; UDP-GlcNAc, uridine-5-diphosphate-*N*-acetylglucosamine; ManNAc, *N*-acetylmannosamine; UDP-ManNAc, uridine-5-diphosphate-*N*-acetylmannosamine; Gro-P, glycerol phosphate; CDP-Gro, cytidyl diphosphate-glycerol; Rbo-P, ribitol phosphate; CDP-Rbo, cytidyl diphosphate-ribitol; ABC, ATP-binding cassette; and LCP, LytR-CpsA-Psr.

Attachment of phages to the outside of the *Staphylococcus* cell (Fig. 2A) is the first stage of infection (Fig. 1). *Staphylococcus* may be resistant to phage adsorption if the receptor molecule is not present, not recognized by the phage, or blocked. Mutations that alter components of the outer surface can have the effect of inhibiting adsorption and, thus, conferring resistance. Through genetic and biochemical studies on a small range of staphylococcal phages, the polyribitol phosphate (poly-RboP) polymer of wall teichoic acid (WTA) or *N*-acetylglucosamine (GlcNAc) modifications at the 4 positions of ribitol phosphate monomers in WTA appear to be the primary targets (35–41).

In an early S. aureus phage resistance study published in 1969, *N*-methyl-*N*′-nitro-*N*-nitrosoguanidine-mutagenized strain H (multilocus sequence type 30 [ST30]) (https://www.phe-culturecollections.org.uk/collections/nctc-3000-project.aspx) phage-resistant mutants were selected by plating on agar plates containing lawns of 52A (siphovirus) (40). Mutants also found to be resistant to phage K (myovirus) were deficient in *N*-acetylglucosamine, cell wall phosphorus, and ester-linked d-alanine in their envelopes, presumably due to a loss of wall teichoic acid production. Further biochemical characterization showed that the mutants lacked UDP-GlcNAc:polyribitol phosphate transferase activity and WTA. Counterintuitively, they did show the relevant biochemical activity for the last known step in WTA biosynthesis (phosphoribitol transferase [TarL]; Fig. 2B) (38). This surprising result suggested that the double-resistant mutants produced ribitol phosphate but either failed to properly polymerize WTA or attach it to the cell wall. These mutants had pleiotropic phenotypic differences from their parent strain (41), including longer generation times than their parent; cell growth in clumps; irregular, rough, gray colonies; and increased levels of wall-bound autolysin. A later study characterizing spontaneous S. aureus strain A170 (ST45) mutants resistant to siphovirus M^Sa^ found similar phenotypic defects (43), and biochemical assays also showed that resistance was likely due to the lack of GlcNAc-modified WTA.

Genes responsible for phage adsorption were identified in a series of elegant molecular genetic studies performed in the RN4220 (ST8) (44) background (35, 36, 45). Deletion of undecaprenyl-phosphate *N*-acetylglucosaminyl 1-phosphate transferase (*tagO*), the first gene involved in WTA biosynthesis, conferred resistance and reduced adsorption to the tested *Myoviridae* (Φ812 and ΦK), while a mutant with a transposon insertion mutation in the *tarM* gene had resistance and reduced adsorption to *Siphoviridae* (ΦSa2mw, Φ47, Φ13, and Φ77). Complementation of wild-type alleles rescued these phenotypes (35). TarM is a glycosyltransferase responsible for attaching α-O-GlcNAc to the 4 position of the ribitol phosphate WTA monomer (46, 47). The *tarM* mutant was previously shown to lack GlcNAc-modified WTA in its envelope (46). TarS, the glycosyltransferase responsible for attaching β-O-GlcNAc to the 4 position of the ribitol phosphate WTA monomer (48), was specifically required for podovirus adsorption (45). Deletion of *tarS* conferred resistance and reduced adsorption to the tested *Podoviridae* (Φ44AHJD, Φ66, and ΦP68) (45), but only deletion of both *tarS* and *tarM* conferred reduced adsorption to the tested *Siphoviridae* (Φ11) in the same RN4220 background used in prior studies (49, 50). On the other hand, even *tarS*^+^ *tarM*^+^ strains were resistant to *Podoviridae*, suggesting that WTA decorated with α-O-GlcNAc by TarM impeded podovirus adsorption (45). Taken together, these findings suggest, for the small number of representatives that were tested, that elimination of WTA confers resistance to all classes of phage, elimination of GlcNAc modifications confers resistance to the *Siphoviridae* and *Podoviridae*, and elimination of the β-O-GlcNAc modification confers resistance specifically to the *Podoviridae*. Given the conservation of wall teichoic acid biosynthesis genes among S. aureus genomes (51) and the cross-species activity of staphylococcal phages, such as phage K (52), these conclusions could be expected to hold in staphylococci beyond S. aureus.

Recent studies have suggested that as the number of strains and phages expands we may find a larger number of genes influencing host range through attachment. Azam et al. conducted a long-term evolution experiment in which they selected S. aureus SA003 (ST352) mutants resistant to myovirus ΦSA012 (53). Resistant mutants gained missense mutations in five genes (*tagO* and the genes for the RNase adapter protein [*rapZ*], putative membrane protein [*yozB*], guanylate kinase [*gmk*], and the alpha subunit of DNA-dependent RNA polymerase [*rpoA*]), a nonsense mutation in one gene (the gene for UDP-*N*-acetylglucosamine 1-carboxyvinyltransferase [*murA2*]), and a 1,779-bp deletion that included the C-terminal region of the teichoic acid glycosyltransferase (*tarS*), a noncoding region, and the N-terminal region of the iron-sulfur repair protein (*scd*). Complementation of the mutations in the genes *scd*, *tagO*, *rapZ*, and *murA2* restored sensitivity to ΦSA012 and adsorption, while only complementation of the mutations in *tarS* restored sensitivity to and adsorption of another myovirus, ΦSA039. The results suggested that while ΦSA012 recognized the WTA backbone, ΦSA039 was unusual in recognizing β-O-GlcNAc-modified WTA, hinting that there may be more variability in receptor targets within phage groups than the limited number of earlier studies suggested.

The carriage of a prophage in certain S. aureus clonal complex 5 (CC5) and CC398 strains that encodes alternative WTA glycosyltransferase (*tarP*) (54) adds further complications. TarP attaches GlcNAc to the 3 position of ribitol phosphate rather than the 4 position, thus conferring sensitivity to *Siphoviridae* (Φ11, Φ52a, and Φ80) but resistance to *Podoviridae* (Φ44, Φ66, and ΦP68). It is interesting in the light of host range evolution that a gene carried on a prophage can change the properties of the S. aureus surface and thus affect the host ranges of other phages.

Although the majority of staphylococcal phages tested bind WTA and GlcNAc receptors, there is one known exception. Siphovirus Φ187 binds WTA glycosylated with *N*-acetyl-d-galactosamine (GalNAc), the unusual WTA synthesized by S. aureus ST395 (55). The genes for α-O-GalNAc transferase (*tagN*), the nucleotide sugar epimerase (*tagV*), and the short GroP WTA polymerase (*tagF*) are required specifically for the synthesis of ST395 WTA. Homologs of these genes were found in the genomes of multiple coagulase-negative *Staphylococcus* (CoNS) strains, such as S. pseudintermedius ED99, S. epidermidis M23864:W1, and S. lugdunensis N920143. Complementation of an S. aureus PS187 C-terminal deletion of the glycosyltransferase (*tagN*) with the wild-type *tagN* gene or that from S. carnosus (*tagN*-Sc) successfully restored the wild-type phenotype, suggesting that *tagN* homologs in other CoNS genomes had functions similar to those in the S. aureus PS187 (ST395) genome. Complementation of the C-terminal deletion of the *tagN* product with either PS187 or S. carnosus *tagN* also restored wild-type sensitivity to Φ187. This difference in WTA structure was shown to prohibit transduction between ST395 and other S. aureus lineages (56). Staphylococcal pathogenicity island (SaPI) particles prepared in an ST1, ST5, ST8, ST22, ST25, or ST30 strain with phage Φ11 or Φ80α failed to be transduced into any ST395 strains. SaPI particles prepared in an ST395 strain, on the other hand, were transduced into other ST395 strains as well as CoNS species and Listeria monocytogenes. These findings suggest that the unique ST395 WTA restricts the phage host range to strains of the same sequence type or Gram-positive bacteria with a related WTA structure, such as Listeria monocytogenes.

There has been one study showing that staphylococcal phages (siphovirus ΦSLT) can bind lipoteichoic acid (LTA), the lipid-anchored, polyglycerol phosphate (GroP) teichoic acid polymer (57) (Fig. 2A). However, subsequent elimination of LTA biosynthesis through *ltaS* deletion had no effect on adsorption of or sensitivity to phage (35), and therefore, the potential significance of LTA as an alternative receptor is currently unknown.

### Effects of surface proteins and extracellular polysaccharides on attachment.

Although proteins serve as receptors for many Gram-positive bacterial phages (for example, the YueB receptor for Bacillus subtilis phage SPP1 [58]), there is no evidence to suggest S. aureus proteins serve as S. aureus phage receptors. Phage interaction protein (Pip) homologs exist throughout the Gram-positive bacteria, serving as protein receptors to which phages irreversibly bind (59). There are Pip surface protein homologs anchored to the staphylococcal cell wall through the action of the sortase enzyme in *Staphylococcus* (60, 61). However, deletion of neither the Pip homologs in RN4220 (ST8) (49) nor sortase A in Newman (ST254) (62, 63) affected sensitivity to phage Φ11 or phages ΦNM1, ΦNM2, and ΦNM4, respectively.

Some classes of proteins or extracellular polysaccharides have been shown to block phage adsorption in the staphylococci through occlusion of the WTA receptors. Overproduction of surface protein A in S. aureus was shown to reduce phage adsorption through this mechanism (64), but work on surface protein occlusion remains limited. Capsule type 1 and 2 strains—strains M (ST1254) (https://www.phe-culturecollections.org.uk/collections/nctc-3000-project.aspx) and Smith diffuse (ST707) (https://www.phe-culturecollections.org.uk/collections/nctc-3000-project.aspx), respectively—were shown to occlude adsorption (65), but strains of the most common capsule types, types 5 and 8, showed inconclusive results (66, 67). Differences in capsule thickness between strains may account for these variable results. Type 1 and 2 strains are mucoid and heavily encapsulated, while type 5 and 8 strains are nonmucoid, despite encapsulation (68). The CoNS species Staphylococcus simulans also showed capsule-dependent inhibition of phage adsorption (69).

The exopolysaccharides (EPS) of staphylococcal biofilms have not been shown to occlude adsorption. Surface proteins, such as biofilm-associated protein (Bap), exopolysaccharides (polysaccharide intercellular adhesin [PIA], composed of poly-*N*-acetylglucosamine [PNAG] and synthesized by the products of the *icaADBC* operon), and extracellular DNA (eDNA) compose staphylococcal biofilms, which can form by PIA-dependent or protein (Bap)-dependent mechanisms (70, 71). Other surface proteins more common than Bap can also mediate biofilm formation, such as FnbA/FnbB (72, 73) and SasG (74) in S. aureus and Aap in S. epidermidis (70). Both S. aureus (19, 75) and S. epidermidis (52, 76, 77) biofilms are susceptible to phage predation. Phage resistance in staphylococcal biofilms may instead be associated with altered biofilm diffusion or metabolism. Studies on S. epidermidis suggested that susceptibility to phages is similar in biofilms and stationary-phase cultures (52). Phages may, in fact, promote bacterial persistence in S. aureus biofilms by releasing nutrients from lysed cells for the remaining live ones to utilize (78).

## BIOSYNTHESIS

### Superinfection immunity.

Staphylococcal temperate phages encode homologs of the cI repressor (17, 18). In Escherichia coli, this protein represses expression of the lytic cycle in newly infecting phages with the same cI protein-binding sites, thus stopping new infections through a mechanism called superinfection immunity. Molecular and evolutionary studies on the E. coli phage lambda model suggest that many superinfection immunity groups (in which member temperate phages confer immunity to each other upon integration) coexist in nature (79), with cI repressor-operator coevolution driving the emergence of new immunity groups (80). Superinfection immunity as a determining factor in phage host range in staphylococcal species appears not to have been studied yet, but since prophages are common (most sequenced S. aureus genomes contain 1 to 4 prophages) (18, 81), it may be a significant barrier to phage infection.

### R-M systems.

Bacteria can resist phage infection by degrading injected phage DNA before it has the chance to replicate and enter the lytic or lysogenic cycle (Fig. 1). Restriction-modification (R-M) is a prominent phage infection barrier in the *Staphylococcus* genus. R-M systems are modular operons containing combinations of host specificity determinant (*hsd*) genes encoding three types of functions: restriction endonuclease activity (*hsdR*), responsible for destroying unmodified DNA; DNA adenine or cytosine methyltransferase activity (*hsdM*), responsible for modifying host DNA so that it is not cleaved by restriction endonucleases; and specificity DNA-binding proteins (*hsdS*), responsible for recognizing sequence motifs targeted for cleavage or modification (82).

There are four known types of R-M systems in bacteria, all of which have been found in the staphylococci (83). In type I systems, the restriction enzyme cleaves unmodified DNA adjacent to its binding site, sometimes separated by as many as 1,000 bp from the binding site, while the modification enzyme methylates host DNA at the target site specified by the specificity protein. A complex containing all three types of subunits restricts unmodified exogenous DNA, while HsdS-HsdM complexes only modify DNA. In type II systems, the restriction enzyme (HsdR_2_) cleaves unmodified DNA at its binding site, while the modification enzyme (HsdM) modifies DNA at this site. In type III systems, the restriction enzyme cleaves unmodified DNA roughly 24 to 28 bp downstream from its asymmetric target site, while the modification enzyme methylates a single strand of host DNA at the target site. The modification subunit (Mod) modifies one strand of DNA either by itself (Mod_2_) or in complex with the restriction subunit (e.g., Mod_2_-Res_1_ or Mod_2_-Res_2_), while the restriction subunit (Res) cleaves unmodified DNA only in complex with modification subunits (Mod_2_-Res_1_ or Mod_2_-Res_2_). In type IV systems, the restriction enzyme cleaves only modified, methylated DNA. Type IV systems do not include a modification enzyme. These systems have been well studied in S. aureus (and in S. epidermidis, to a more limited extent) due to their role in restricting natural horizontal gene transfer and genetic manipulation of the organism (83–86).

Type I R-M systems are the most abundant class of R-M systems reported in S. aureus, followed by type IV and then type II systems (83). Type III systems appear to be rare, with only two being described in the genus (83). Analyses of the restriction enzyme genomic database REBASE in 2014 showed that all completed S. aureus genomes encode a type I R-M system and that most S. aureus genomes annotated with R-M genes encode a type I system (83, 87). The most common type I R-M locus found in S. aureus is Sau1 (88). Expression of a functional Sau1 *hsdR* gene in restriction-deficient S. aureus strain RN4220 greatly reduced electroporation, conjugation, and transduction frequencies (88). S. aureus genomes generally encode two Sau1 *hsdS* genes that specify two distinct DNA motif targets for restriction or modification (89). The Sau1 HsdS subunit determines target specificity through its two target recognition domains (TRDs), each of which binds to one part of the target sequence (90). TRDs are the least conserved portions of the HsdS amino acid sequences (88) and vary in carriage between strains with lineage- and/or clonal complex-specific variant associations, as microarray hybridization studies indicate (88, 89). The Sau1 system prevented the transfer of plasmid DNA from one clonal complex (CC5) to another (CC8) with a different target recognition site (89), showing that restriction defines barriers between clonal complexes. Sau1 also affected the susceptibility of two CC8 strains (strains NCTC8325-4 and RN4220 p*hsdR*) but not the *hsdR*-deficient strain RN4220 to phage Φ75 (siphovirus) propagated in a CC51 strain (879R4RF), suggesting that Sau1 can control phage host range (88). Sau1 variation is a powerful marker of the lineage/clonal complex (88, 91) and likely drives the independent evolution of clonal complexes. Sau1 would therefore be predicted to be a major host range limitation to phages grown in a strain of a different clonal complex. Since the target sites of nearly all S. aureus Sau1 R-M systems from each of the different clonal complexes have now been identified (90), it should be possible to bioinformatically predict the Sau1-defined clonal complex host range of any sequenced bacteriophage.

The type IV R-M system SauUSI is estimated to be found in 90% of S. aureus strains (83, 92) and, in combination with Sau1, presents an effective restriction barrier for resisting phage infection (93). SauUSI specifically restricts DNA methylated or hydroxymethylated at the C-5 position of cytosine (92). The preferred binding site for SauUSI is Sm5CNGS, where S represents either cytosine or guanine (92). Type II R-M systems have been estimated to be present in ∼33% of strains and display a range of target sites (83, 94–96). The most common type II R-M system found in S. aureus is called Sau3A (94). The Sau3A restriction enzyme cleaves 5′ to the guanine in unmodified 5′-GATC-3′ sequences. The Sau3A modification enzyme, on the other hand, methylates the restriction site at the C-5 position of cytosine (97). Some type II systems, such as Sau42I, are encoded by phages. Sau42I is an example of a type IIS R-M system, which binds asymmetric DNA sequences and cleaves outside the recognition site, unlike most type II systems (82). Unlike type I and type IV systems, type II systems are often carried on mobile genetic elements, which are capable of frequent transfer between strains and which are not conserved among all members of the same clonal complex, so they present a more strain-specific and variable limit to host range (87). Certain S. aureus type II R-M systems (e.g., Sau96I) serve to negate the type IV SauUSI system because they methylate cytosines and guanines in sequences that SauUSI targets for cleavage. This is an interesting example of how R-M systems acquired by HGT can have unpredictable interactions with existing systems.

If unmodified phages can survive restriction enzyme degradation upon cell entry, the phage DNA molecules acquire protective DNA methylation as they replicate. While survival of restriction can happen stochastically at high multiplicities of infection, phages have also been shown to have evolved or acquired adaptations for restriction evasion. Antirestriction mechanisms include restriction site alteration, restriction site occlusion, indirect subversion of restriction-modification activity, and direct inhibition of restriction-modification systems (98). Restriction site alteration can include both incorporation of alternative bases, such as 5-hydroxymethyluracil (5hmU) and 5-hydroxymethylcytosine (5hmC), and loss of restriction sites through selection. A clear example of the latter in the staphylococci is the elimination of GATC sites in the 140-kb phage K genome, enabling its avoidance of Sau3A restriction (99). Another example is the evolution of particular antimicrobial resistance-carrying conjugative plasmids which have lost specific Sau1 R-M sites, allowing their transfer between common MRSA lineages (88). Restriction site occlusion refers to DNA-binding proteins preventing restriction enzymes from binding and digesting DNA (98, 100, 101). R-M subversion occurs through either stimulation of host modification enzymes or destruction of restriction cofactors (e.g., *S*-adenosylmethionine [SAM]) (98, 102, 103). R-M inhibition occurs most often in type I systems (but also in some type II systems) through the binding of specific antirestriction proteins, such as ArdA, ArdB, and Ocr (98, 104, 105). There is no literature specifically characterizing antirestriction in *Staphylococcus*, but an E. coli *ardA* homolog has been identified in the staphylococcal Tn*916* and Tn*5801* transposons (106).

### CRISPR systems.

Clustered regularly interspaced short palindromic repeats (CRISPRs) confer immunity to phage infection through the cleavage of extrinsic DNA in a sequence-specific manner. Unlike R-M systems, which target specific DNA sequence motifs, CRISPRs adaptively incorporate target sequences from phages that they have destroyed to increase the efficiency of protection. After integrating short segments of foreign DNA as spacers of CRISPR arrays, CRISPR-associated (Cas) nucleases process the transcribed CRISPR array RNA into CRISPR RNAs (crRNAs), used to target new incursions of identical foreign DNA elements for destruction (107, 108). Surveys of S. aureus and S. epidermidis genomes indicate that CRISPRs are not common in these species (109, 110). These surveys looked for the presence of the *cas6* and *cas9* genes, which encode nucleases required for type I/III and type II CRISPR-mediated resistance, respectively. Cas6 is an endoribonuclease found in type I and III CRISPR systems that cleaves pre-crRNA transcripts within the 3′ end of the repeat region to produce mature guide crRNAs (111, 112), while Cas9 is an endonuclease found in type II CRISPR systems that cleaves DNA in a crRNA-guided manner (112, 113). Only 12 of 300 published S. epidermidis genomes searched encoded the Cas6 nuclease, 18 of 130 S. epidermidis isolates from Denmark (Copenhagen University Hospital) tested positive for *cas6* via PCR, and 14 of nearly 5,000 published S. aureus genomes encoded CRISPR/Cas systems (109). Another study specifically examining S. aureus found that 2 of 32 S. aureus strains encoded CRISPR-Cas systems (110). These CRISPRs were similar to those found in two S. lugdunensis strains, suggesting that they were recombined with S. lugdunensis or derived from a common ancestor (110). CRISPR/Cas systems have also occasionally been reported in strains of other species (S. capitis, S. schleiferi, S. intermedius, S. argenteus, and S. microti) (109). Only a single S. aureus strain has been reported to encode Cas9, which is found in a staphylococcal cassette chromosome *mec* element (SCC*mec*)-like region (114). Nonetheless, CRISPR systems have been shown to be important in resisting the introduction of foreign DNA in S. epidermidis RP62a (115, 116). Anti-CRISPR mechanisms, such as proteins that prevent CRISPR-Cas systems from binding DNA target sites, are being discovered in many phages (117–119), although they have not yet been discovered in those specific for staphylococci. Currently discovered anti-CRISPR mechanisms have been shown to target both type I and type II CRISPR systems (117–120).

## ASSEMBLY

Assembly interference is the parasitization of superinfecting phage by chromosomal phage-like elements and has been demonstrated experimentally in S. aureus pathogenicity island (SaPI)-helper phage interactions. SaPIs encode important virulence factors, such as toxic shock syndrome toxin (TSST), but are mobilized only by superinfecting helper siphoviruses (28, 121). The Dut dUTPase protein expressed by helper phages derepresses the Stl SaPI repressor, activating the SaPI lytic cycle (28). The derepressed SaPIs then take advantage of the superinfection to proliferate at the expense of the helper phage. SaPIs interfere with helper phage assembly through several mechanisms (122): remodeling phage capsid proteins to fit the small SaPI genome (123–127), encoding phage packaging interference (Ppi) proteins that prevent helper phage DNA packaging into new SaPI particles (123), and disrupting phage late gene activation (128). All known SaPIs encode phage packaging interference (Ppi) proteins, which divert phage DNA packaging toward SaPIs by inhibiting helper phage terminase small subunits (TerS_P_) but not the corresponding SaPI subunits (TerS_S_) (123). Ppi proteins are divided into two classes based on sequences that differ in helper phage specificity. Class I interferes with Φ80α and Φ11, while class II interferes with Φ12 (123). The PtiM-modulated PtiA and the PtiB SaPI2 proteins inhibit expression of the LtrC-activated phage 80 late gene operon (packaging and lysis genes), thus interfering with later steps of the helper phage life cycle (128). The SaPI particles then go on to infect new S. aureus hosts, integrating their DNA into the chromosome instead of killing the cell. Helper phages and SaPIs are thought to gain and lose resistance to each other in a Red Queen scenario, given the observed rapid coevolution of their respective *dut* and *stl* genes (129). SaPIs are found throughout *Staphylococcus* species and beyond; therefore, they may be a common strain-specific modifier of siphovirus infection potential.

## OTHER PHAGE HOST RANGE-LIMITING FACTORS

Several uncommon or less well understood mechanisms may contribute to phage host range limitation in *Staphylococcus*. One abortive infection (Abi) system, the eukaryote-like serine/threonine kinase Stk2, has been characterized in S. aureus and S. epidermidis (130). In this case, siphovirus infection results in self-induced killing of the host cell, preventing the amplification and spread of phages in the population. Stk2 was found to be activated by a phage protein of unknown function and caused cell death by phosphorylating host proteins involved in diverse core cellular functions. Only S. epidermidis RP62A and a few S. aureus strains encode Stk2, however, suggesting limited genus-wide importance. The recent long-term evolution study on S. aureus strain SA003 uncovered two genes involved in postadsorption resistance to myovirus ΦSA012 (53). Missense mutations in guanylate kinase and the alpha subunit of DNA-dependent RNA polymerase conferred resistance but not corresponding decreases in the adsorption rate, suggesting some postadsorption role in resisting infection. More phage resistance systems likely remain undiscovered. A genome-wide association study of 207 clinical MRSA strains and 12 phage preparations identified 167 gene families putatively associated with phage-bacterium interactions (131). While these families included restriction-modification genes, transcriptional regulators, and genes of prophage and SaPI origin, most were accessory gene families of unknown function.

## PHAGE HOST RANGE IN *STAPHYLOCOCCUS* IS DETERMINED BY A HIERARCHICAL COMBINATION OF HOST FACTORS

**FIG 3:**
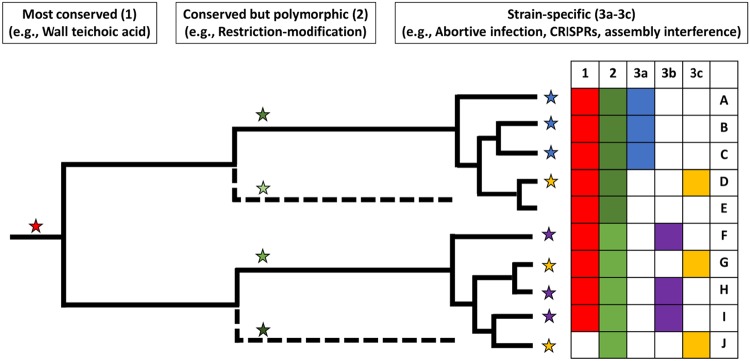
Phage host range for an individual strain is the combination of multiple factors that have different levels of conservation within the species. This is illustrated by a hypothetical phylogenetic tree. Mechanisms can be present throughout strains (1, most conserved; red), present in many strains but with considerable allelic variation (2, conserved but polymorphic; shades of green), or present in a few strains, possibly with allelic variation (3a to 3c, less conserved with potential polymorphism; blue, purple, and yellow, respectively). Branches where mechanisms evolved by mutation or homologous recombination, in the case of mechanisms 1 and 2, or were acquired by HGT, in the case of mechanisms 3a to 3c, are annotated with colored stars. The table on the right summarizes the mechanisms (1 to 3c) present in each strain (strains A to J) using shaded boxes with corresponding colors. Strain J has a mutation that results in the null phenotype for the red mechanism. Host range is the result of the combination of mechanisms present, so strains A to C as well as F, H, and I would be predicted to have identical host ranges, but phage-specific factors could also introduce variability.

In summary, we have described how the host range of a *Staphylococcus* phage is determined by a combination of both host- and phage-encoded genes, as well as the epigenetic DNA methylation patterns conferred on its DNA from the last strain that it infected. Bacterium-encoded factors can be conceived of as affecting host range at different levels within the species (Fig. 3). At the highest level, most phages’ target for receptor binding (WTA) is highly conserved across *Staphylococcus* species. Strains with unusual WTAs, such as S. aureus ST395 and CoNS strains with poly-GroP WTA (55, 56), would be expected to be genetically isolated within the genus. Type I R-M *hsdS* allotypes and capsule type are conserved between most strains of the same clonal complex (CC) but differ between isolates of different CC groups and thus contribute to defining the host range in a large subset of S. aureus strains. At the level of individual strains, inserted prophages and SaPIs, Stk2, type II systems acquired by HGT, and other as yet unknown functions may all serve to limit the host range. We know even less about phage-encoded systems that counteract host resistance. The finding that lytic phages (*Myoviridae* and *Podoviridae*) tend to have broader host ranges than *Siphoviridae* when challenged against the same set of *Staphylococcus* strains suggests that the former encode an array of uncharacterized genes that work against host defenses.

## FUTURE DIRECTIONS

Although much progress has been made in the past 5 decades toward understanding the mechanisms that define staphylococcal phage host range, numerous important questions remain. We need to know more about species other than S. aureus and S. epidermidis, and even within these species, we need to make sure that rarer and non-methicillin-resistant strains are included in studies (132). We also need to ensure that our collections reflect the true diversity of phages that infect *Staphylococcus* species. Even within the two main species, only a relatively small number of phages have been tested. This will lead us to consider the questions of phage ecology when understanding what types of phages are found in different environments and with what abundance.

Discovering novel phage resistance mechanisms would aid the effort to understand determinants of host range. Many phage resistance mechanisms have been identified and characterized in other Gram-positive bacteria and other bacteria generally but not in the staphylococci. Superinfection exclusion (Sie) and abortive infection (Abi) systems, for example, are well characterized in the lactococci (133–135). In addition, a recent publication describes some 26 new antiphage defense systems identified in bacteria (136), not including the recently discovered bacteriophage exclusion (BREX) and defense island system associated with restriction-modification (DISARM) phage defenses (137–139). Five of the 10 verified, newly discovered antiphage defense systems (Thoeris, Hachiman, Gabija, Lamassu, and Kiwa) have orthologs in staphylococcal genomes (136).

Understanding phage host range to the point that we can make accurate predictions based on the host genome will be important for developing phage therapies against *Staphylococcus* strains. Ideally, cocktail formulations for therapy consist of phages with broad, nonoverlapping host ranges against the target species (or clonal complex) to be treated. As there are many more genome sequences available than strains that can be tested for sensitivity in the laboratory (e.g., >40,000 for S. aureus) (140), with a predictive model we could run *in silico* tests on genome sequences to model the efficacy of the cocktail. With the potential for genome sequencing to be used in the future as a primary clinical diagnostic, we could modify the cocktail to contain phages that specifically target the bacterium causing the infection.

Knowledge of phage host range will also lead us to understand the fitness costs of resistance and its potential trade-offs with the virulence and antibiotic resistance of *Staphylococcus*. Strains with null mutations in biosynthetic genes are rare, given WTA’s roles in cell division, autolysis, virulence, and antibiotic resistance (36, 37). Although knocking out the genes involved in the first two steps of WTA biosynthesis has no fitness cost in S. aureus (at least under laboratory conditions) (141, 142), WTA has many critical physiological roles, especially in environments subject to phage therapy. Staphylococcal WTA is required for nasal colonization (141, 143), cell division (41, 43), regulating autolysis (144, 145), lysozyme resistance through cell wall cross-linking (132, 146), resistance to cationic antimicrobial peptides and fatty acids (147, 148), and biofilm formation (149). WTA-altered or -negative phage-resistant mutants would, in turn, become less virulent (43) and even antibiotic sensitive, which would make them highly unfit in the natural habitat colonizing mammalian hosts or in an infection site subject to treatment. Given that methicillin resistance requires WTA (50), phage–beta-lactam combination therapies could be particularly promising. Mutants resistant to either phages or beta-lactams would be sensitive to the other treatment, assuming that the infecting strain is sensitive to the phage treatment. Nonetheless, as we note for host range, strains containing minor but fitness-neutral resistance mechanisms, such as R-M systems, may be the most recalcitrant to phage therapy. Staphylococcal phage therapies must then overcome both immediate, emerging mutational resistance and intrinsic resistance mechanisms (e.g., R-M systems) specific to strains or clonal complexes. These resistance limitations, however, could be overcome by selecting phage host range mutants that escaped host resistance mechanisms, thus isolating more useful phages that would form more effective phage cocktails (150, 151).

The phage-resistant mutants isolated so far, such as those described in the adsorption studies, were typically selected in rich, aerated laboratory medium. The consequences for fitness of the same mutations occurring during *in vivo* infection might be more severe. In addition, both the relevance of various resistance mechanisms *in vivo* and the effect of strain genetic background on resistance selection, especially on a species-wide scale, have been left unexamined in most previous work. One study in mammalian hosts showed that the environment altered phage transfer frequency and selection (152), leading to the spread of prophage and the selection of phage resistance by superimmunity. In laboratory media, the phage transfer frequency was lower and the spread of prophage was less pronounced (152). It will be important to know both how quickly and in which loci mutations emerge, as well as the more general distribution of resistance gene families.

Finally, it is interesting to consider what phage host range studies reveal about the hosts themselves. Staphylococci seem to be unusual among Gram-positive bacteria in requiring conserved WTA receptors for attachment and having no reported role for protein receptors. Differences in the outer surface of *Staphylococcus* and/or a feature of the phage ecology within the genus may account for this fact. Another interesting question is why CRISPRs play a lesser role for intercepting extrinsic phage DNA than R-M systems in this genus compared with others. It could be that CRISPR systems have a finite capacity for carrying fragments of mobile genetic elements, while R-M systems can attack a wider range of incoming DNA, relevant to rapidly evolving populations. Future studies that probe these questions may reveal some of the differential evolutionary forces that shape the genomes of pathogenic bacteria.

## CONCLUSIONS

Staphylococcal phage resistance mechanisms have been identified at three stages of infection (attachment, biosynthesis, and assembly) and regulate host range in a hierarchical manner, depending on mechanism conservation. We need further studies to objectively identify the contribution of individual phage resistance mechanisms to host range. Such work would provide the information needed not only to formulate phage cocktails effective against a wide variety of strains but also to overcome remaining obstacles to cocktail development (e.g., highly effective R-M or Abi systems). Future studies relevant to phage therapy should also characterize phage resistance development during infection and therapy as well as the effects of resistance on mutant fitness. Taken together, this future work will inform the rational design of phage cocktails to treat staphylococcal infections alone or in combination with antibiotics.
